# (*E*)-2-Hydr­oxy-6-[(4-propyl­phenyl)­iminiometh­yl]phenolate

**DOI:** 10.1107/S1600536809052246

**Published:** 2009-12-09

**Authors:** Serap Yazıcı, Çiğdem Albayrak, İsmail Gümrükçüoğlu, İsmet Şenel, Orhan Büyükgüngör

**Affiliations:** a Department of Physics, Faculty of Arts and Sciences, Ondokuz Mayıs University, TR-55139, Kurupelit-Samsun, Turkey; b Faculty of Education, Sinop University, TR-57000 Sinop, Turkey; c Department of Chemistry, Ondokuz Mayıs University, TR-55139, Kurupelit-Samsun, Turkey

## Abstract

The title compound, C_16_H_17_NO_2_, crystallizes with three crystallographically independent zwitterionic mol­ecules in the asymmetric unit which differ significantly in the orientations of the propyl side chains. The dihedral angles between the two benzene rings in the three mol­ecules are 6.17 (7), 6.75 (7) and 23.67 (7)°, respectively. In each independent mol­ecule, an intra­molecular N—H⋯O hydrogen bond generates an *S*(6) ring motif. In the crystal, each independent mol­ecule exists as part of an O—H⋯O hydrogen-bonded centrosymmetric *R*_2_^2^(10) dimer.

## Related literature

For general background to Schiff base compounds in coordination chemistry, see: Cohen *et al.* (1964 ▶); Moustakali-Mavridis *et al.* (1978 ▶); Hadjoudis *et al.* (1987 ▶); Ogawa & Harada (2003 ▶); Krygowski *et al.* (1997 ▶). For related structures, see: Petek *et al.* (2006 ▶); Kılıç *et al.* (2008 ▶); Gao *et al.* (2005 ▶); Temel *et al.* (2006 ▶). For bond-length data, see: Allen *et al.* (1987 ▶). 
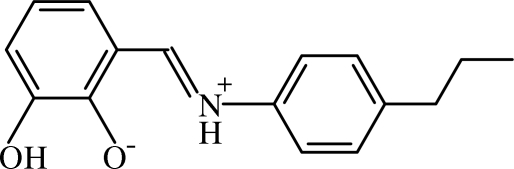


## Experimental

### 

#### Crystal data

C_16_H_17_NO_2_
*M* *_r_* = 255.31
Triclinic, 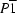

*a* = 11.5743 (4) Å
*b* = 12.7635 (4) Å
*c* = 14.1706 (5) Å
α = 90.418 (3)°
β = 103.259 (3)°
γ = 95.540 (3)°
*V* = 2027.14 (11) Å^3^
*Z* = 6
Mo *K*α radiation
μ = 0.08 mm^−1^
*T* = 150 K
0.56 × 0.42 × 0.28 mm

#### Data collection

Stoe IPDS II diffractometer
Absorption correction: integration (*X-RED32*; Stoe & Cie, 2002 ▶) *T*_min_ = 0.972, *T*_max_ = 0.985
25371 measured reflections
7976 independent reflections
6241 reflections with *I* > 2σ(*I*)
*R*_int_ = 0.028

#### Refinement

*R*[*F* ^2^ > 2σ(*F* ^2^)] = 0.042
*wR*(*F* ^2^) = 0.110
*S* = 1.04
7976 reflections
538 parameters
H atoms treated by a mixture of independent and constrained refinement
Δρ_max_ = 0.33 e Å^−3^
Δρ_min_ = −0.26 e Å^−3^

### 

Data collection: *X-AREA* (Stoe & Cie, 2002 ▶); cell refinement: *X-AREA*; data reduction: *X-RED32* (Stoe & Cie, 2002 ▶); program(s) used to solve structure: *SHELXS97* (Sheldrick, 2008 ▶); program(s) used to refine structure: *SHELXL97* (Sheldrick, 2008 ▶); molecular graphics: *ORTEP-3 for Windows* (Farrugia, 1997 ▶); software used to prepare material for publication: *WinGX* (Farrugia, 1999 ▶).

## Supplementary Material

Crystal structure: contains datablocks I, global. DOI: 10.1107/S1600536809052246/ci2970sup1.cif

Structure factors: contains datablocks I. DOI: 10.1107/S1600536809052246/ci2970Isup2.hkl

Additional supplementary materials:  crystallographic information; 3D view; checkCIF report

## Figures and Tables

**Table 1:** Hydrogen-bond geometry (Å, °)

*D*—H⋯*A*	*D*—H	H⋯*A*	*D*⋯*A*	*D*—H⋯*A*
N1*A*—H1*A*⋯O1*A*	1.02 (2)	1.63 (2)	2.5444 (16)	147 (2)
N1*B*—H1*B*⋯O1*B*	1.06 (2)	1.57 (2)	2.5229 (16)	146 (2)
N1*C*—H1*C*⋯O1*C*	1.08 (2)	1.57 (2)	2.5502 (17)	149 (2)
O2*A*—H5*A*⋯O1*A*^i^	0.90 (2)	1.89 (2)	2.7032 (17)	148 (2)
O2*B*—H5*B*⋯O1*B*^ii^	0.89 (2)	1.91 (2)	2.7049 (15)	148 (2)
O2*C*—H5*C*⋯O1*C*^iii^	0.92 (2)	1.84 (2)	2.6659 (15)	148 (2)

Symmetry codes: (i) 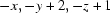; (ii) 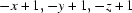; (iii) 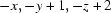.

**Fig. 1.**
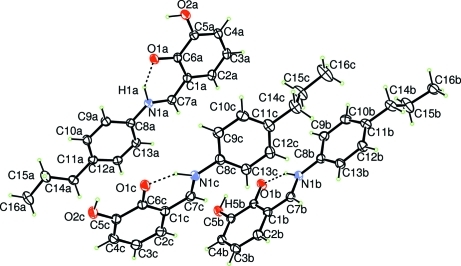
The three independent molecules of the title compound, with the atomic numbering scheme. Displacement ellipsoids are drawn at the 30% probability level.

**Fig. 2.**
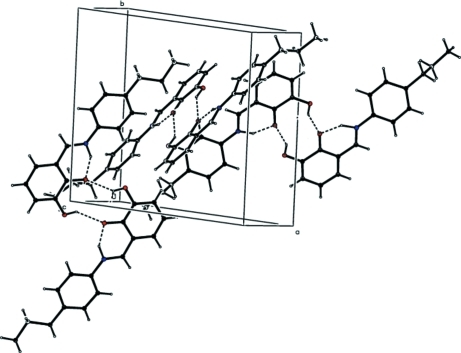
A partial packing diagram of the title compound. Hydrogen bonds are shown as dashed lines.

## supplementary crystallographic information

### Comment

Schiff bases are of interest because they have long been known to show photochromism and thermochromism in the solid state which may involve reversible proton transfer from the amino N atom to the hydroxyl O atom (Cohen *et al.*, 1964; Moustakali-Mavridis *et al.*, 1978; Hadjoudis *et al.*, 1987). On the basis of some thermochromic and photochromic Schiff base compounds, it was proposed that molecules exhibiting thermochromism are planar, while those exhibiting photochromism are non-planar (Moustakali-Mavridis *et al.*, 1978). In general, there are two types of tautomeric forms in Schiff bases *viz*. keto-amine (quinoid with N—H···O bond) and phenol-imine (benzenoid with O—H···N bond). Quinoid tautomers can also be found in the zwitterionic form which consist of ionic intramolecular N^+^—H···O^-^ hydrogen bond (Ogawa & Harada, 2003). The zwitterionic form is rarely seen in the solid state (Krygowski *et al.*, 1997).

The three independent molecules (A, B and C) of the title compound are shown in Fig. 1. The N^+^—H bond lengths in molecules A, B and C are [1.02 (2), 1.06 (2) and 1.08 (3) Å, respectively] as expected in the zwitterionic form of Schiff bases (Petek *et al.*, 2006; Kılıç *et al.*, 2008). These values are longer than the N—H distance of 0.87 Å. The C6—O1 bond lengths in molecules A, B and C are 1.3034 (18), 1.3028 (17), 1.3060 (18) Å, respectively, and are intermediate between C—O single (1.362 Å) and C═O double bond (1.222 Å) lengths (Allen *et al.*, 1987). The C7—N1 bond lengths [1.3034 (19), 1.3068 (19) and 1.3065 (19) Å for A, B and C, respectively] are comparable to those observed in related zwitterions (Gao *et al.*,2005; Temel *et al.*, 2006). Each molecule displays an E configuration with respect to its C═N bond. The dihedral angle between the two benzene rings in molecules A, B and C are 6.17 (7)°, 6.75 (7)° and 23.67 (7)°, respectively. In each independent molecule, an intramolecular N—H ···O hydrogen bond generates an S(6) ring motif (Table 1).

The crystal packing is stabilized by intermolecular O—H···O hydrogen bonds (Table 1), generating centrosymmetric *R*_2_^2^ (10) dimers (Fig.2).

### Experimental

The title compound was prepared by refluxing a mixture of 2,3-dihydroxybenzaldehyde (0.5 g 3.6 mmol) in ethanol (20 ml) and 4-propylaniline (0.49 g 3.6 mmol) in ethanol (20 ml). The reaction mixture was stirred for 1 h under reflux. Single crystals of the title compound suitable for X-ray analysis were obtained by slow evaporation of an ethanol solution (yield 68%, m.p. 371–372 K).

### Refinement

N- and O-bound H atoms were located in a difference map and refined freely. C-bound H atoms were placed in calculated positions and constrained to ride on their parents atoms, with C–H = 0.93–0.99 Å and *U*_iso_(H) = 1.2*U*_eq_(C) and 1.5*U*_eq_(C_methyl_).

### Crystal data

**Table:**

C_16_H_17_NO_2_	*Z* = 6
*M**_r_* = 255.31	*F*(000) = 816
Triclinic, *P*1	*D*_x_ = 1.255 Mg m^−^^3^
Hall symbol: -P 1	Mo *K*α radiation, λ = 0.71073 Å
*a* = 11.5743 (4) Å	Cell parameters from 36582 reflections
*b* = 12.7635 (4) Å	θ = 1.6–28.0°
*c* = 14.1706 (5) Å	µ = 0.08 mm^−^^1^
α = 90.418 (3)°	*T* = 150 K
β = 103.259 (3)°	Prism, dark red
γ = 95.540 (3)°	0.56 × 0.42 × 0.28 mm
*V* = 2027.14 (11) Å^3^	

### Data collection

**Table:**

Stoe IPDS II diffractometer	7976 independent reflections
Radiation source: fine-focus sealed tube	6241 reflections with *I* > 2σ(*I*)
graphite	*R*_int_ = 0.028
Detector resolution: 6.67 pixels mm^-1^	θ_max_ = 26.0°, θ_min_ = 1.6°
ω scans	*h* = −14→14
Absorption correction: integration (*X-RED32*; Stoe & Cie, 2002)	*k* = −15→15
*T*_min_ = 0.972, *T*_max_ = 0.985	*l* = −16→17
25371 measured reflections	

### Refinement

**Table:**

Refinement on *F*^2^	Primary atom site location: structure-invariant direct methods
Least-squares matrix: full	Secondary atom site location: difference Fourier map
*R*[*F*^2^ > 2σ(*F*^2^)] = 0.042	Hydrogen site location: inferred from neighbouring sites
*wR*(*F*^2^) = 0.110	H atoms treated by a mixture of independent and constrained refinement
*S* = 1.04	*w* = 1/[σ^2^(*F*_o_^2^) + (0.0592*P*)^2^ + 0.2353*P*] where *P* = (*F*_o_^2^ + 2*F*_c_^2^)/3
7976 reflections	(Δ/σ)_max_ = 0.003
538 parameters	Δρ_max_ = 0.33 e Å^−^^3^
0 restraints	Δρ_min_ = −0.26 e Å^−^^3^

### Special details

**Table:**

Experimental. 316 frames, detector distance = 100 mm
Geometry. All e.s.d.'s (except the e.s.d. in the dihedral angle between two l.s. planes) are estimated using the full covariance matrix. The cell e.s.d.'s are taken into account individually in the estimation of e.s.d.'s in distances, angles and torsion angles; correlations between e.s.d.'s in cell parameters are only used when they are defined by crystal symmetry. An approximate (isotropic) treatment of cell e.s.d.'s is used for estimating e.s.d.'s involving l.s. planes.
Refinement. Refinement of *F*^2^ against ALL reflections. The weighted *R*-factor *wR* and goodness of fit *S* are based on *F*^2^, conventional *R*-factors *R* are based on *F*, with *F* set to zero for negative *F*^2^. The threshold expression of *F*^2^ > σ(*F*^2^) is used only for calculating *R*-factors(gt) *etc*. and is not relevant to the choice of reflections for refinement. *R*-factors based on *F*^2^ are statistically about twice as large as those based on *F*, and *R*- factors based on ALL data will be even larger.

### Fractional atomic coordinates and isotropic or equivalent isotropic displacement parameters (Å^2^)

**Table:**

	*x*	*y*	*z*	*U*_iso_*/*U*_eq_	
C15B	1.2012 (2)	0.92858 (17)	0.75163 (17)	0.0717 (6)	
H15C	1.1547	0.9385	0.6867	0.086*	
H15D	1.2324	0.8608	0.7514	0.086*	
C16C	0.8117 (3)	1.0067 (2)	0.87450 (19)	0.0926 (8)	
H16A	0.8187	1.0279	0.8110	0.139*	
H16B	0.8864	0.9853	0.9097	0.139*	
H16C	0.7910	1.0649	0.9085	0.139*	
C1C	0.19899 (13)	0.38530 (11)	0.85202 (11)	0.0367 (3)	
C2C	0.18638 (15)	0.29139 (12)	0.79542 (12)	0.0436 (4)	
H2C	0.2466	0.2765	0.7653	0.052*	
C3C	0.08639 (15)	0.22263 (12)	0.78489 (12)	0.0464 (4)	
H3C	0.0789	0.1607	0.7481	0.056*	
C4C	−0.00539 (15)	0.24484 (12)	0.82929 (11)	0.0434 (4)	
H4C	−0.0737	0.1977	0.8209	0.052*	
C5C	0.00403 (13)	0.33476 (12)	0.88471 (11)	0.0385 (3)	
C6C	0.10763 (13)	0.40783 (11)	0.89947 (10)	0.0356 (3)	
C7C	0.30005 (14)	0.45906 (12)	0.85868 (11)	0.0371 (3)	
C8C	0.41020 (13)	0.62687 (11)	0.92008 (10)	0.0353 (3)	
C9C	0.39200 (14)	0.72868 (12)	0.94441 (11)	0.0411 (3)	
H9C	0.3171	0.7436	0.9509	0.049*	
C10C	0.48500 (16)	0.80766 (13)	0.95891 (11)	0.0458 (4)	
H10C	0.4717	0.8755	0.9750	0.055*	
C11C	0.59814 (15)	0.78809 (13)	0.94994 (11)	0.0446 (4)	
C12C	0.61472 (15)	0.68592 (13)	0.92674 (12)	0.0453 (4)	
H12C	0.6899	0.6709	0.9210	0.054*	
C13C	0.52270 (14)	0.60541 (12)	0.91182 (11)	0.0416 (3)	
H13C	0.5363	0.5374	0.8964	0.050*	
C14C	0.69818 (18)	0.87517 (15)	0.96110 (13)	0.0579 (5)	
H14E	0.7716	0.8494	0.9964	0.069*	
H14K	0.6814	0.9328	0.9993	0.069*	
C15C	0.7159 (2)	0.91595 (16)	0.86547 (15)	0.0693 (6)	
H15E	0.6411	0.9382	0.8290	0.083*	
H15K	0.7361	0.8588	0.8287	0.083*	
N1C	0.31247 (11)	0.54844 (9)	0.90723 (9)	0.0364 (3)	
O1C	0.11620 (9)	0.49230 (8)	0.95420 (8)	0.0419 (2)	
O2C	−0.08724 (9)	0.35566 (9)	0.92583 (8)	0.0444 (3)	
C1B	0.63614 (13)	0.42043 (11)	0.74837 (10)	0.0335 (3)	
C2B	0.60523 (14)	0.34026 (12)	0.80990 (11)	0.0411 (3)	
H2B	0.6482	0.3393	0.8739	0.049*	
C3B	0.51316 (14)	0.26497 (12)	0.77577 (11)	0.0420 (3)	
H3B	0.4933	0.2132	0.8168	0.050*	
C4B	0.44776 (13)	0.26496 (11)	0.67878 (11)	0.0365 (3)	
H4B	0.3846	0.2135	0.6564	0.044*	
C5B	0.47644 (12)	0.34010 (11)	0.61725 (10)	0.0333 (3)	
C6B	0.57181 (12)	0.42046 (10)	0.65011 (10)	0.0320 (3)	
C7B	0.72796 (13)	0.50219 (11)	0.78451 (10)	0.0345 (3)	
C8B	0.84359 (13)	0.66407 (11)	0.75582 (10)	0.0335 (3)	
C9B	0.84990 (14)	0.74062 (11)	0.68709 (11)	0.0387 (3)	
H9B	0.7952	0.7354	0.6275	0.046*	
C10B	0.93783 (15)	0.82436 (12)	0.70778 (12)	0.0445 (4)	
H10B	0.9415	0.8749	0.6614	0.053*	
C11B	1.02074 (15)	0.83477 (11)	0.79620 (11)	0.0416 (3)	
C12B	1.01168 (14)	0.75811 (12)	0.86405 (11)	0.0421 (3)	
H12B	1.0654	0.7640	0.9241	0.051*	
C13B	0.92505 (14)	0.67347 (12)	0.84472 (11)	0.0392 (3)	
H13B	0.9214	0.6230	0.8912	0.047*	
C14B	1.11991 (17)	0.92395 (13)	0.81796 (13)	0.0517 (4)	
H14C	1.0845	0.9900	0.8156	0.062*	
H14D	1.1663	0.9173	0.8836	0.062*	
C16B	1.30486 (17)	1.01232 (13)	0.77300 (14)	0.0547 (4)	
H16K	1.3518	1.0070	0.7257	0.082*	
H16L	1.3532	1.0031	0.8366	0.082*	
H16M	1.2758	1.0804	0.7704	0.082*	
N1B	0.75502 (10)	0.57790 (9)	0.72934 (9)	0.0333 (3)	
O1B	0.59829 (9)	0.49057 (8)	0.59003 (7)	0.0402 (2)	
O2B	0.41492 (9)	0.33925 (9)	0.52316 (7)	0.0400 (2)	
H1A	0.023 (2)	0.7711 (18)	0.4586 (16)	0.085 (7)*	
H1B	0.701 (2)	0.5644 (18)	0.6580 (17)	0.089 (7)*	
H1C	0.235 (2)	0.5505 (19)	0.9370 (18)	0.097 (8)*	
H5A	0.101 (2)	1.0706 (16)	0.5252 (15)	0.072 (7)*	
H5B	0.440 (2)	0.3985 (18)	0.4978 (16)	0.079 (7)*	
H5C	−0.069 (2)	0.4157 (19)	0.9650 (18)	0.087 (8)*	
H7A	0.1778 (16)	0.6796 (14)	0.3642 (12)	0.050 (5)*	
H7B	0.7716 (15)	0.4995 (12)	0.8521 (12)	0.044 (4)*	
H7C	0.3628 (15)	0.4447 (12)	0.8263 (11)	0.037 (4)*	
C1A	0.20614 (13)	0.83448 (11)	0.41888 (10)	0.0362 (3)	
C2A	0.31409 (14)	0.85933 (13)	0.38903 (12)	0.0443 (4)	
H2A	0.3450	0.8081	0.3578	0.053*	
C3A	0.37295 (14)	0.95801 (13)	0.40592 (13)	0.0472 (4)	
H3A	0.4439	0.9737	0.3864	0.057*	
C4A	0.32645 (14)	1.03589 (12)	0.45274 (12)	0.0424 (4)	
H4A	0.3676	1.1027	0.4645	0.051*	
C5A	0.22182 (13)	1.01489 (11)	0.48125 (11)	0.0374 (3)	
C6A	0.15744 (13)	0.91300 (11)	0.46528 (10)	0.0351 (3)	
C7A	0.14494 (13)	0.73221 (11)	0.40063 (11)	0.0371 (3)	
C8A	−0.02951 (13)	0.61121 (10)	0.40566 (10)	0.0339 (3)	
C9A	−0.13686 (14)	0.60527 (11)	0.43360 (11)	0.0393 (3)	
H9A	−0.1566	0.6624	0.4656	0.047*	
C10A	−0.21506 (14)	0.51438 (11)	0.41402 (11)	0.0398 (3)	
H10A	−0.2867	0.5111	0.4336	0.048*	
C11A	−0.18835 (13)	0.42820 (11)	0.36572 (10)	0.0347 (3)	
C12A	−0.08046 (14)	0.43637 (11)	0.33756 (11)	0.0402 (3)	
H12A	−0.0612	0.3798	0.3045	0.048*	
C13A	−0.00149 (14)	0.52570 (11)	0.35715 (12)	0.0409 (3)	
H13A	0.0704	0.5289	0.3380	0.049*	
C14A	−0.27040 (14)	0.32742 (11)	0.34356 (11)	0.0393 (3)	
H14A	−0.2310	0.2721	0.3807	0.047*	
H14B	−0.2804	0.3089	0.2755	0.047*	
C15A	−0.39285 (16)	0.32782 (13)	0.36385 (13)	0.0509 (4)	
H15A	−0.4341	0.3821	0.3266	0.061*	
H15B	−0.3846	0.3448	0.4321	0.061*	
C16A	−0.46688 (18)	0.22241 (15)	0.33830 (15)	0.0619 (5)	
H16D	−0.5437	0.2259	0.3524	0.093*	
H16E	−0.4270	0.1686	0.3758	0.093*	
H16F	−0.4767	0.2061	0.2705	0.093*	
N1A	0.04501 (11)	0.70683 (9)	0.42637 (9)	0.0357 (3)	
O1A	0.05774 (9)	0.89431 (8)	0.49299 (8)	0.0427 (3)	
O2A	0.17798 (11)	1.09159 (8)	0.52600 (9)	0.0464 (3)	

### Atomic displacement parameters (Å^2^)

**Table:**

	*U*^11^	*U*^22^	*U*^33^	*U*^12^	*U*^13^	*U*^23^
C15B	0.0710 (14)	0.0682 (12)	0.0782 (14)	−0.0263 (11)	0.0370 (11)	−0.0294 (11)
C16C	0.105 (2)	0.0791 (15)	0.0895 (17)	−0.0477 (14)	0.0382 (15)	−0.0118 (13)
C1C	0.0338 (8)	0.0373 (7)	0.0392 (8)	0.0047 (6)	0.0083 (6)	0.0088 (6)
C2C	0.0433 (9)	0.0405 (8)	0.0486 (9)	0.0061 (7)	0.0136 (7)	0.0053 (7)
C3C	0.0518 (10)	0.0365 (8)	0.0495 (9)	0.0035 (7)	0.0094 (7)	0.0032 (7)
C4C	0.0409 (9)	0.0398 (8)	0.0455 (9)	−0.0043 (7)	0.0047 (7)	0.0108 (7)
C5C	0.0333 (8)	0.0430 (8)	0.0390 (8)	0.0034 (6)	0.0077 (6)	0.0139 (6)
C6C	0.0334 (8)	0.0363 (7)	0.0361 (7)	0.0031 (6)	0.0060 (6)	0.0092 (6)
C7C	0.0332 (8)	0.0426 (8)	0.0375 (7)	0.0067 (6)	0.0106 (6)	0.0088 (6)
C8C	0.0357 (8)	0.0395 (7)	0.0313 (7)	0.0000 (6)	0.0102 (6)	0.0073 (6)
C9C	0.0419 (9)	0.0437 (8)	0.0402 (8)	0.0051 (7)	0.0138 (7)	0.0039 (6)
C10C	0.0568 (10)	0.0404 (8)	0.0410 (8)	0.0000 (7)	0.0147 (7)	0.0008 (6)
C11C	0.0493 (10)	0.0500 (9)	0.0317 (7)	−0.0097 (7)	0.0094 (7)	0.0024 (6)
C12C	0.0372 (9)	0.0536 (9)	0.0456 (9)	−0.0023 (7)	0.0135 (7)	0.0030 (7)
C13C	0.0380 (9)	0.0416 (8)	0.0466 (9)	0.0023 (6)	0.0133 (7)	0.0035 (6)
C14C	0.0588 (12)	0.0574 (11)	0.0528 (10)	−0.0184 (9)	0.0135 (9)	−0.0063 (8)
C15C	0.0825 (15)	0.0599 (11)	0.0639 (12)	−0.0278 (11)	0.0281 (11)	−0.0048 (9)
N1C	0.0330 (7)	0.0389 (6)	0.0388 (7)	0.0022 (5)	0.0118 (5)	0.0074 (5)
O1C	0.0356 (6)	0.0443 (6)	0.0485 (6)	0.0006 (5)	0.0166 (5)	0.0010 (5)
O2C	0.0328 (6)	0.0509 (7)	0.0493 (6)	−0.0021 (5)	0.0114 (5)	0.0057 (5)
C1B	0.0319 (7)	0.0347 (7)	0.0348 (7)	0.0040 (6)	0.0092 (6)	0.0026 (6)
C2B	0.0420 (9)	0.0448 (8)	0.0352 (8)	0.0008 (7)	0.0076 (6)	0.0074 (6)
C3B	0.0439 (9)	0.0394 (8)	0.0438 (8)	−0.0015 (7)	0.0143 (7)	0.0095 (6)
C4B	0.0329 (8)	0.0343 (7)	0.0435 (8)	−0.0006 (6)	0.0134 (6)	−0.0006 (6)
C5B	0.0302 (7)	0.0368 (7)	0.0343 (7)	0.0048 (6)	0.0099 (6)	−0.0021 (6)
C6B	0.0307 (7)	0.0335 (7)	0.0342 (7)	0.0046 (6)	0.0115 (6)	0.0028 (5)
C7B	0.0321 (8)	0.0388 (7)	0.0324 (7)	0.0035 (6)	0.0068 (6)	0.0028 (6)
C8B	0.0314 (7)	0.0338 (7)	0.0358 (7)	0.0028 (6)	0.0088 (6)	0.0007 (6)
C9B	0.0407 (8)	0.0409 (8)	0.0345 (7)	0.0053 (6)	0.0079 (6)	0.0043 (6)
C10B	0.0541 (10)	0.0357 (8)	0.0454 (9)	0.0032 (7)	0.0155 (7)	0.0102 (6)
C11B	0.0475 (9)	0.0336 (7)	0.0447 (8)	−0.0024 (6)	0.0157 (7)	−0.0009 (6)
C12B	0.0425 (9)	0.0436 (8)	0.0372 (8)	−0.0049 (7)	0.0065 (6)	0.0007 (6)
C13B	0.0398 (8)	0.0393 (8)	0.0363 (7)	−0.0027 (6)	0.0068 (6)	0.0067 (6)
C14B	0.0614 (11)	0.0386 (8)	0.0553 (10)	−0.0092 (8)	0.0200 (8)	−0.0031 (7)
C16B	0.0539 (11)	0.0424 (9)	0.0711 (12)	−0.0034 (8)	0.0245 (9)	0.0001 (8)
N1B	0.0303 (6)	0.0358 (6)	0.0331 (6)	0.0009 (5)	0.0068 (5)	0.0017 (5)
O1B	0.0414 (6)	0.0421 (5)	0.0337 (5)	−0.0057 (4)	0.0060 (4)	0.0062 (4)
O2B	0.0393 (6)	0.0429 (6)	0.0346 (5)	−0.0061 (5)	0.0062 (4)	−0.0002 (4)
C1A	0.0305 (8)	0.0379 (7)	0.0393 (8)	0.0026 (6)	0.0068 (6)	0.0032 (6)
C2A	0.0334 (8)	0.0466 (9)	0.0552 (9)	0.0050 (7)	0.0150 (7)	0.0001 (7)
C3A	0.0309 (8)	0.0517 (9)	0.0603 (10)	−0.0007 (7)	0.0155 (7)	0.0040 (8)
C4A	0.0335 (8)	0.0393 (8)	0.0512 (9)	−0.0036 (6)	0.0061 (7)	0.0044 (7)
C5A	0.0347 (8)	0.0351 (7)	0.0403 (8)	0.0012 (6)	0.0056 (6)	0.0012 (6)
C6A	0.0298 (7)	0.0364 (7)	0.0380 (7)	0.0014 (6)	0.0062 (6)	0.0029 (6)
C7A	0.0332 (8)	0.0372 (7)	0.0407 (8)	0.0047 (6)	0.0078 (6)	−0.0006 (6)
C8A	0.0340 (8)	0.0307 (7)	0.0361 (7)	0.0026 (6)	0.0063 (6)	0.0011 (5)
C9A	0.0419 (9)	0.0346 (7)	0.0442 (8)	0.0020 (6)	0.0167 (7)	−0.0059 (6)
C10A	0.0373 (8)	0.0390 (8)	0.0453 (8)	−0.0004 (6)	0.0160 (7)	−0.0022 (6)
C11A	0.0373 (8)	0.0335 (7)	0.0324 (7)	0.0037 (6)	0.0063 (6)	0.0030 (5)
C12A	0.0412 (9)	0.0318 (7)	0.0492 (9)	0.0068 (6)	0.0125 (7)	−0.0033 (6)
C13A	0.0341 (8)	0.0373 (8)	0.0542 (9)	0.0056 (6)	0.0157 (7)	−0.0013 (7)
C14A	0.0446 (9)	0.0359 (7)	0.0358 (7)	−0.0013 (6)	0.0086 (6)	−0.0010 (6)
C15A	0.0488 (10)	0.0472 (9)	0.0567 (10)	−0.0099 (8)	0.0186 (8)	−0.0079 (8)
C16A	0.0590 (12)	0.0602 (11)	0.0645 (12)	−0.0228 (9)	0.0223 (9)	−0.0141 (9)
N1A	0.0341 (7)	0.0320 (6)	0.0409 (7)	0.0019 (5)	0.0095 (5)	−0.0002 (5)
O1A	0.0377 (6)	0.0362 (5)	0.0581 (7)	−0.0026 (4)	0.0218 (5)	−0.0048 (5)
O2A	0.0415 (7)	0.0352 (5)	0.0632 (7)	−0.0043 (5)	0.0172 (5)	−0.0069 (5)

### Geometric parameters (Å, °)

**Table:**

C15B—C14B	1.472 (3)	C8B—C9B	1.393 (2)
C15B—C16B	1.502 (2)	C8B—N1B	1.4146 (18)
C15B—H15C	0.97	C9B—C10B	1.383 (2)
C15B—H15D	0.97	C9B—H9B	0.93
C16C—C15C	1.508 (3)	C10B—C11B	1.389 (2)
C16C—H16A	0.96	C10B—H10B	0.93
C16C—H16B	0.96	C11B—C12B	1.390 (2)
C16C—H16C	0.96	C11B—C14B	1.513 (2)
C1C—C7C	1.414 (2)	C12B—C13B	1.381 (2)
C1C—C2C	1.414 (2)	C12B—H12B	0.93
C1C—C6C	1.426 (2)	C13B—H13B	0.93
C2C—C3C	1.362 (2)	C14B—H14C	0.97
C2C—H2C	0.93	C14B—H14D	0.97
C3C—C4C	1.402 (2)	C16B—H16K	0.96
C3C—H3C	0.93	C16B—H16L	0.96
C4C—C5C	1.367 (2)	C16B—H16M	0.96
C4C—H4C	0.93	N1B—H1B	1.06 (2)
C5C—O2C	1.3642 (19)	O2B—H5B	0.89 (2)
C5C—C6C	1.420 (2)	C1A—C7A	1.416 (2)
C6C—O1C	1.3060 (18)	C1A—C2A	1.417 (2)
C7C—N1C	1.3065 (19)	C1A—C6A	1.423 (2)
C7C—H7C	0.974 (17)	C2A—C3A	1.365 (2)
C8C—C13C	1.387 (2)	C2A—H2A	0.93
C8C—C9C	1.390 (2)	C3A—C4A	1.405 (2)
C8C—N1C	1.4139 (19)	C3A—H3A	0.93
C9C—C10C	1.380 (2)	C4A—C5A	1.366 (2)
C9C—H9C	0.93	C4A—H4A	0.93
C10C—C11C	1.390 (2)	C5A—O2A	1.3611 (18)
C10C—H10C	0.93	C5A—C6A	1.424 (2)
C11C—C12C	1.384 (2)	C6A—O1A	1.3034 (18)
C11C—C14C	1.505 (2)	C7A—N1A	1.3034 (19)
C12C—C13C	1.385 (2)	C7A—H7A	0.998 (18)
C12C—H12C	0.93	C8A—C9A	1.384 (2)
C13C—H13C	0.93	C8A—C13A	1.389 (2)
C14C—C15C	1.505 (3)	C8A—N1A	1.4125 (18)
C14C—H14E	0.97	C9A—C10A	1.385 (2)
C14C—H14K	0.97	C9A—H9A	0.93
C15C—H15E	0.97	C10A—C11A	1.388 (2)
C15C—H15K	0.97	C10A—H10A	0.93
N1C—H1C	1.08 (3)	C11A—C12A	1.390 (2)
O2C—H5C	0.92 (2)	C11A—C14A	1.509 (2)
C1B—C7B	1.417 (2)	C12A—C13A	1.375 (2)
C1B—C6B	1.4205 (19)	C12A—H12A	0.93
C1B—C2B	1.421 (2)	C13A—H13A	0.93
C2B—C3B	1.361 (2)	C14A—C15A	1.510 (2)
C2B—H2B	0.93	C14A—H14A	0.97
C3B—C4B	1.409 (2)	C14A—H14B	0.97
C3B—H3B	0.93	C15A—C16A	1.518 (2)
C4B—C5B	1.369 (2)	C15A—H15A	0.97
C4B—H4B	0.93	C15A—H15B	0.97
C5B—O2B	1.3593 (17)	C16A—H16D	0.96
C5B—C6B	1.423 (2)	C16A—H16E	0.96
C6B—O1B	1.3028 (17)	C16A—H16F	0.96
C7B—N1B	1.3068 (19)	N1A—H1A	1.02 (2)
C7B—H7B	0.977 (17)	O2A—H5A	0.91 (2)
C8B—C13B	1.386 (2)		
			
C14B—C15B—C16B	117.01 (16)	C10B—C9B—H9B	120.1
C14B—C15B—H15C	108.0	C8B—C9B—H9B	120.1
C16B—C15B—H15C	108.0	C9B—C10B—C11B	121.61 (14)
C14B—C15B—H15D	108.0	C9B—C10B—H10B	119.2
C16B—C15B—H15D	108.0	C11B—C10B—H10B	119.2
H15C—C15B—H15D	107.3	C10B—C11B—C12B	117.52 (14)
C15C—C16C—H16A	109.5	C10B—C11B—C14B	121.90 (14)
C15C—C16C—H16B	109.5	C12B—C11B—C14B	120.55 (15)
H16A—C16C—H16B	109.5	C13B—C12B—C11B	121.84 (14)
C15C—C16C—H16C	109.5	C13B—C12B—H12B	119.1
H16A—C16C—H16C	109.5	C11B—C12B—H12B	119.1
H16B—C16C—H16C	109.5	C12B—C13B—C8B	119.78 (14)
C7C—C1C—C2C	120.06 (14)	C12B—C13B—H13B	120.1
C7C—C1C—C6C	119.77 (13)	C8B—C13B—H13B	120.1
C2C—C1C—C6C	120.12 (14)	C15B—C14B—C11B	114.85 (14)
C3C—C2C—C1C	120.24 (15)	C15B—C14B—H14C	108.6
C3C—C2C—H2C	119.9	C11B—C14B—H14C	108.6
C1C—C2C—H2C	119.9	C15B—C14B—H14D	108.6
C2C—C3C—C4C	120.29 (15)	C11B—C14B—H14D	108.6
C2C—C3C—H3C	119.9	H14C—C14B—H14D	107.5
C4C—C3C—H3C	119.9	C15B—C16B—H16K	109.5
C5C—C4C—C3C	120.98 (15)	C15B—C16B—H16L	109.5
C5C—C4C—H4C	119.5	H16K—C16B—H16L	109.5
C3C—C4C—H4C	119.5	C15B—C16B—H16M	109.5
O2C—C5C—C4C	119.98 (14)	H16K—C16B—H16M	109.5
O2C—C5C—C6C	119.21 (14)	H16L—C16B—H16M	109.5
C4C—C5C—C6C	120.80 (14)	C7B—N1B—C8B	127.37 (12)
O1C—C6C—C5C	120.01 (13)	C7B—N1B—H1B	109.2 (13)
O1C—C6C—C1C	122.46 (13)	C8B—N1B—H1B	123.4 (13)
C5C—C6C—C1C	117.53 (13)	C5B—O2B—H5B	107.0 (14)
N1C—C7C—C1C	121.75 (14)	C7A—C1A—C2A	120.17 (14)
N1C—C7C—H7C	117.8 (9)	C7A—C1A—C6A	119.76 (13)
C1C—C7C—H7C	120.5 (9)	C2A—C1A—C6A	120.05 (13)
C13C—C8C—C9C	119.41 (14)	C3A—C2A—C1A	120.36 (15)
C13C—C8C—N1C	122.68 (13)	C3A—C2A—H2A	119.8
C9C—C8C—N1C	117.88 (13)	C1A—C2A—H2A	119.8
C10C—C9C—C8C	120.04 (15)	C2A—C3A—C4A	120.10 (15)
C10C—C9C—H9C	120.0	C2A—C3A—H3A	119.9
C8C—C9C—H9C	120.0	C4A—C3A—H3A	119.9
C9C—C10C—C11C	121.47 (15)	C5A—C4A—C3A	120.99 (14)
C9C—C10C—H10C	119.3	C5A—C4A—H4A	119.5
C11C—C10C—H10C	119.3	C3A—C4A—H4A	119.5
C12C—C11C—C10C	117.58 (15)	O2A—C5A—C4A	120.07 (13)
C12C—C11C—C14C	120.88 (16)	O2A—C5A—C6A	119.07 (13)
C10C—C11C—C14C	121.50 (16)	C4A—C5A—C6A	120.86 (14)
C11C—C12C—C13C	121.96 (15)	O1A—C6A—C1A	122.32 (13)
C11C—C12C—H12C	119.0	O1A—C6A—C5A	120.06 (13)
C13C—C12C—H12C	119.0	C1A—C6A—C5A	117.63 (13)
C12C—C13C—C8C	119.53 (15)	N1A—C7A—C1A	121.83 (14)
C12C—C13C—H13C	120.2	N1A—C7A—H7A	119.6 (10)
C8C—C13C—H13C	120.2	C1A—C7A—H7A	118.5 (10)
C11C—C14C—C15C	112.95 (15)	C9A—C8A—C13A	119.41 (13)
C11C—C14C—H14E	109.0	C9A—C8A—N1A	117.20 (12)
C15C—C14C—H14E	109.0	C13A—C8A—N1A	123.37 (13)
C11C—C14C—H14K	109.0	C8A—C9A—C10A	120.16 (13)
C15C—C14C—H14K	109.0	C8A—C9A—H9A	119.9
H14E—C14C—H14K	107.8	C10A—C9A—H9A	119.9
C14C—C15C—C16C	114.12 (18)	C9A—C10A—C11A	121.14 (14)
C14C—C15C—H15E	108.7	C9A—C10A—H10A	119.4
C16C—C15C—H15E	108.7	C11A—C10A—H10A	119.4
C14C—C15C—H15K	108.7	C10A—C11A—C12A	117.65 (13)
C16C—C15C—H15K	108.7	C10A—C11A—C14A	122.92 (13)
H15E—C15C—H15K	107.6	C12A—C11A—C14A	119.43 (13)
C7C—N1C—C8C	126.57 (13)	C13A—C12A—C11A	121.90 (13)
C7C—N1C—H1C	107.1 (13)	C13A—C12A—H12A	119.1
C8C—N1C—H1C	126.3 (13)	C11A—C12A—H12A	119.1
C5C—O2C—H5C	112.3 (15)	C12A—C13A—C8A	119.73 (14)
C7B—C1B—C6B	119.51 (13)	C12A—C13A—H13A	120.1
C7B—C1B—C2B	120.87 (13)	C8A—C13A—H13A	120.1
C6B—C1B—C2B	119.60 (13)	C11A—C14A—C15A	117.08 (13)
C3B—C2B—C1B	120.45 (14)	C11A—C14A—H14A	108.0
C3B—C2B—H2B	119.8	C15A—C14A—H14A	108.0
C1B—C2B—H2B	119.8	C11A—C14A—H14B	108.0
C2B—C3B—C4B	120.46 (14)	C15A—C14A—H14B	108.0
C2B—C3B—H3B	119.8	H14A—C14A—H14B	107.3
C4B—C3B—H3B	119.8	C14A—C15A—C16A	112.08 (15)
C5B—C4B—C3B	120.54 (14)	C14A—C15A—H15A	109.2
C5B—C4B—H4B	119.7	C16A—C15A—H15A	109.2
C3B—C4B—H4B	119.7	C14A—C15A—H15B	109.2
O2B—C5B—C4B	120.72 (13)	C16A—C15A—H15B	109.2
O2B—C5B—C6B	118.48 (12)	H15A—C15A—H15B	107.9
C4B—C5B—C6B	120.80 (13)	C15A—C16A—H16D	109.5
O1B—C6B—C1B	122.10 (13)	C15A—C16A—H16E	109.5
O1B—C6B—C5B	119.75 (12)	H16D—C16A—H16E	109.5
C1B—C6B—C5B	118.15 (12)	C15A—C16A—H16F	109.5
N1B—C7B—C1B	121.44 (13)	H16D—C16A—H16F	109.5
N1B—C7B—H7B	120.8 (10)	H16E—C16A—H16F	109.5
C1B—C7B—H7B	117.7 (10)	C7A—N1A—C8A	127.46 (13)
C13B—C8B—C9B	119.43 (13)	C7A—N1A—H1A	108.7 (13)
C13B—C8B—N1B	123.12 (13)	C8A—N1A—H1A	123.5 (13)
C9B—C8B—N1B	117.42 (13)	C5A—O2A—H5A	108.3 (13)
C10B—C9B—C8B	119.80 (14)		
			
C7C—C1C—C2C—C3C	−176.72 (15)	C13B—C8B—C9B—C10B	0.7 (2)
C6C—C1C—C2C—C3C	0.8 (2)	N1B—C8B—C9B—C10B	−177.44 (13)
C1C—C2C—C3C—C4C	0.6 (2)	C8B—C9B—C10B—C11B	−0.2 (2)
C2C—C3C—C4C—C5C	−0.8 (2)	C9B—C10B—C11B—C12B	−0.6 (2)
C3C—C4C—C5C—O2C	178.86 (13)	C9B—C10B—C11B—C14B	177.71 (15)
C3C—C4C—C5C—C6C	−0.5 (2)	C10B—C11B—C12B—C13B	1.0 (2)
O2C—C5C—C6C—O1C	2.0 (2)	C14B—C11B—C12B—C13B	−177.33 (15)
C4C—C5C—C6C—O1C	−178.63 (13)	C11B—C12B—C13B—C8B	−0.6 (2)
O2C—C5C—C6C—C1C	−177.47 (12)	C9B—C8B—C13B—C12B	−0.3 (2)
C4C—C5C—C6C—C1C	1.9 (2)	N1B—C8B—C13B—C12B	177.72 (14)
C7C—C1C—C6C—O1C	−4.0 (2)	C16B—C15B—C14B—C11B	−176.99 (18)
C2C—C1C—C6C—O1C	178.48 (13)	C10B—C11B—C14B—C15B	−59.4 (2)
C7C—C1C—C6C—C5C	175.51 (13)	C12B—C11B—C14B—C15B	118.8 (2)
C2C—C1C—C6C—C5C	−2.1 (2)	C1B—C7B—N1B—C8B	179.71 (13)
C2C—C1C—C7C—N1C	177.33 (14)	C13B—C8B—N1B—C7B	7.4 (2)
C6C—C1C—C7C—N1C	−0.2 (2)	C9B—C8B—N1B—C7B	−174.52 (14)
C13C—C8C—C9C—C10C	0.8 (2)	C7A—C1A—C2A—C3A	179.57 (15)
N1C—C8C—C9C—C10C	179.19 (13)	C6A—C1A—C2A—C3A	1.1 (2)
C8C—C9C—C10C—C11C	−0.1 (2)	C1A—C2A—C3A—C4A	−0.3 (3)
C9C—C10C—C11C—C12C	−0.6 (2)	C2A—C3A—C4A—C5A	−0.6 (2)
C9C—C10C—C11C—C14C	177.07 (15)	C3A—C4A—C5A—O2A	−179.63 (14)
C10C—C11C—C12C—C13C	0.6 (2)	C3A—C4A—C5A—C6A	0.7 (2)
C14C—C11C—C12C—C13C	−177.05 (15)	C7A—C1A—C6A—O1A	0.6 (2)
C11C—C12C—C13C—C8C	0.1 (2)	C2A—C1A—C6A—O1A	179.17 (14)
C9C—C8C—C13C—C12C	−0.8 (2)	C7A—C1A—C6A—C5A	−179.45 (13)
N1C—C8C—C13C—C12C	−179.07 (13)	C2A—C1A—C6A—C5A	−0.9 (2)
C12C—C11C—C14C—C15C	79.4 (2)	O2A—C5A—C6A—O1A	0.3 (2)
C10C—C11C—C14C—C15C	−98.2 (2)	C4A—C5A—C6A—O1A	179.97 (14)
C11C—C14C—C15C—C16C	177.3 (2)	O2A—C5A—C6A—C1A	−179.61 (13)
C1C—C7C—N1C—C8C	178.85 (13)	C4A—C5A—C6A—C1A	0.1 (2)
C13C—C8C—N1C—C7C	−23.0 (2)	C2A—C1A—C7A—N1A	−179.46 (14)
C9C—C8C—N1C—C7C	158.72 (14)	C6A—C1A—C7A—N1A	−0.9 (2)
C7B—C1B—C2B—C3B	176.83 (14)	C13A—C8A—C9A—C10A	0.6 (2)
C6B—C1B—C2B—C3B	−1.3 (2)	N1A—C8A—C9A—C10A	178.91 (13)
C1B—C2B—C3B—C4B	0.5 (2)	C8A—C9A—C10A—C11A	−0.5 (2)
C2B—C3B—C4B—C5B	0.5 (2)	C9A—C10A—C11A—C12A	−0.1 (2)
C3B—C4B—C5B—O2B	178.82 (13)	C9A—C10A—C11A—C14A	179.53 (14)
C3B—C4B—C5B—C6B	−0.8 (2)	C10A—C11A—C12A—C13A	0.6 (2)
C7B—C1B—C6B—O1B	3.6 (2)	C14A—C11A—C12A—C13A	−179.01 (14)
C2B—C1B—C6B—O1B	−178.32 (13)	C11A—C12A—C13A—C8A	−0.6 (2)
C7B—C1B—C6B—C5B	−177.09 (12)	C9A—C8A—C13A—C12A	0.0 (2)
C2B—C1B—C6B—C5B	1.0 (2)	N1A—C8A—C13A—C12A	−178.28 (14)
O2B—C5B—C6B—O1B	−0.26 (19)	C10A—C11A—C14A—C15A	8.2 (2)
C4B—C5B—C6B—O1B	179.34 (13)	C12A—C11A—C14A—C15A	−172.20 (14)
O2B—C5B—C6B—C1B	−179.63 (12)	C11A—C14A—C15A—C16A	179.78 (14)
C4B—C5B—C6B—C1B	0.0 (2)	C1A—C7A—N1A—C8A	174.36 (13)
C6B—C1B—C7B—N1B	0.0 (2)	C9A—C8A—N1A—C7A	−174.64 (14)
C2B—C1B—C7B—N1B	−178.13 (14)	C13A—C8A—N1A—C7A	3.6 (2)

### Hydrogen-bond geometry (Å, °)

**Table:**

*D*—H···*A*	*D*—H	H···*A*	*D*···*A*	*D*—H···*A*
N1A—H1A···O1A	1.02 (2)	1.63 (2)	2.5444 (16)	147 (2)
N1B—H1B···O1B	1.06 (2)	1.57 (2)	2.5229 (16)	146 (2)
N1C—H1C···O1C	1.08 (2)	1.57 (2)	2.5502 (17)	149 (2)
O2A—H5A···O1A^i^	0.90 (2)	1.89 (2)	2.7032 (17)	148 (2)
O2B—H5B···O1B^ii^	0.89 (2)	1.91 (2)	2.7049 (15)	148 (2)
O2C—H5C···O1C^iii^	0.92 (2)	1.84 (2)	2.6659 (15)	148 (2)

Symmetry codes: (i) −*x*, −*y*+2, −*z*+1; (ii) −*x*+1, −*y*+1, −*z*+1; (iii) −*x*, −*y*+1, −*z*+2.
